# Changes in Anti-Thyroglobulin IgG Glycosylation Patterns in Hashimoto's Thyroiditis Patients

**DOI:** 10.1210/jc.2014-2921

**Published:** 2014-11-07

**Authors:** Shanshan Yuan, Qianqian Li, Yang Zhang, Chuncui Huang, Hongmei Wu, Yan Li, Yalei Liu, Nan Yu, Hong Zhang, Guizhi Lu, Yanming Gao, Ying Gao, Xiaohui Guo

**Affiliations:** Department of Endocrinology (S.Y., Y.Z., Yal.L., N.Y., H.Z., G.L., Ya.G., Yi.G., X.G.), Peking University First Hospital, Beijing 100034, China; Institute of Biophysics (Q.L., C.H., H.W., Yan.L.), Chinese Academy of Sciences, Beijing 100101, China

## Abstract

**Objective:**

Sera of Hashimoto's thyroiditis (HT) patients are known to exhibit elevated levels of anti-thyroglobulin IgG (TgAb IgG). Therefore, TgAb IgG represents a hallmark of this debilitating autoimmune disease. The aim of our study was to investigate the differential expression of specific glycosylation patterns of TgAb IgG from HT patients and healthy blood donors.

**Methods:**

HT patients (n = 32) were divided into two subgroups, medium level group (mHT, n = 15) and high level group (hHT, n = 17), according to the serum levels of TgAb detected by electrochemiluminescence immunoassay. TgAb IgG was purified by affinity chromatography from the sera of the HT group and control group (n = 15). MALDI-QIT-TOF-MS/MS spectrometry was performed to identify the glycosylation profiles of purified TgAb IgG. Lectin microarray technology was used to compare the abundance of different glycans found on TgAb IgG between HT patients and controls, and between the mHT and hHT groups.

**Results:**

The results by MALDI-QIT-TOF-MS/MS showed that the glycosylation profiles of TgAb IgG were similar between the mHT, hHT, and control groups. Furthermore, the lectin microarray showed that compared to the control group (all *P* < .001), there were higher levels present of (1) mannose (detected as lectin LCA, VFA, and MNA-M); (2) terminal sialic acid (detected as SNA-I and PSA); (3) core fucose (detected as LcH); and (4) Gal(β1–4)GlcNAc(β1–2)Man glycans (detected as PHA-L) on TgAb IgG from the HT group. A similar trend was observed between the hHT and mHT group, with elevated levels of mannose, terminal sialic acid, core fucose, and Gal(β1–4)GlcNAc(β1–2)Man glycans on TgAb IgG found in the hHT group compared with the mHT group (all *P* < .05).

**Conclusions:**

TgAb IgG of HT patients exhibits higher glycosylation levels than those observed for TgAb IgG of healthy controls. Our results provide new clues for exploring the role of TgAb in the pathogenesis of HT.

Hashimoto's thyroiditis (HT), an autoimmune thyroid disease, is one of the most widespread thyroid disorders. It is characterized by a diffuse goiter, lymphocytic infiltration in the thyroid tissue, and the presence of thyroid auto-antibodies in the sera of HT patients. The incidence rate of HT has recently increased so far for unknown reasons, and has reached 0.3–1.5 cases per 1000 population every year (1). HT is the most common cause of hypothyroidism, a condition that seriously affects the growth and development of children, in addition to lowering the quality of life (QOL) of adults. HT exhibits a complex etiology, which is currently incompletely understood. Thus, investigating the etiology of HT is paramount for the prevention and treatment of hypothyroidism.

Serum antithyroglobulin antibody (TgAb) is one of hallmarks of HT, where it reaches elevated levels in 80–90% of all HT patients (2). In healthy individuals, TgAbs are only present in serum at low levels (3, 4). In vitro experiments have shown that TgAb had an effect on antibody-dependent cellular cytotoxicity (ADCC), which indicated that it might be involved in thyrocyte destruction (5). TgAb predominantly consists of antibodies of the IgG class (6). IgG antibodies are glycoproteins, which on average contain 2.8 N-linked glycans per protein molecule. Two N-linked glycans are invariably located at asparagine 297 of the Fc region of the two heavy chains, and additional N-linked glycans are found within the Fab region (7). The two N-linked glycans within the Fc region were shown to play an important role not only in the structure but also in the Fc-mediated biological function of IgGs (8). Therefore, investigating the glycosylation patterns and levels of TgAb IgG in the sera of HT patients may help to better understand the biological role of TgAb in the pathogenesis of HT.

Glycosylation is one of the most widespread modifications found in proteins, and is considered to greatly affect a number of different protein functions, such as protein-protein interactions, cell-cell recognition, adhesion, and motility (9–12). Alterations of the glycosylation patterns of IgG have been found in many kinds of autoimmune diseases (13–15). It has been found that the level of IgG galactosylation is decreased in rheumatoid arthritis, and the decrease is related to the degree of the disease (16–18). In addition, our previous study showed that the glycosylation patterns of sera TgAb IgG varied in different thyroid diseases. Also, the sialic acid content on TgAb IgG was negatively correlated with serum TgAb IgG levels in patients (19). Together, these results indicate that changes in the glycosylation pattern on TgAb might be involved in the pathogenesis of thyroid diseases. Therefore, in order to expand our current understanding of the pathogenesis of HT disease, we focused on investigating the alterations to the TgAb glycosylation patterns in HT patients.

Among the recently developed technologies for glycomic analysis, two methods have been established to analyze the protein glycosylation with high sensitivity and throughput (20). Tandem mass spectrometry (MS/MS) allows for powerful sequence analysis of N-linked carbohydrate chain (< 40 monosaccharide) of glycoprotein, only requiring minute amounts of glycan sample of approximately 50–100 ng. In this method, N-linked carbohydrate chains are released from glycoproteins by endoglycosidases. The method has been successfully applied to the analysis of glycosylation patterns of proteins from serum and tissue samples of patients. The lectin microarray, in contrast, is based on the interaction of glycans with different glycan-binding proteins, such as lectins and antibodies, and has been developed to analyze glycosylation patterns in a quantitative manner. Even though it can only detect accessible carbohydrate motifs, and not the entire repertoire of glycoforms (21), lectin microarray is a sensitive and high-throughput platform that can detect and verify glycosylation changes from biosamples without requirement for sample pretreatment which usually needs only 1–5 μL serum for glycosylation detection and identification. Both methods are comparable to glycosylation analysis both in terms of sensitivity and ability for high sample throughput.

Thus, the aim of our study was to identify the glycosylation patterns of TgAb IgG in the sera of HT patients, and their changes relative to those found in sera of healthy blood donors. To this end, we used matrix-assisted laser desorption/ionization quadrupole ion trap time-of-flight mass spectrometry (MALDI-QIT-TOF-MS/MS) and high-density lectin microarray to detect the TgAb IgG glycosylation patterns.

## Materials and Methods

### Subjects

This study was approved by the Ethics Committee of Peking University First Hospital, and all participants gave informed written consent. A total of 32 HT patients diagnosed prior to this study with elevated TgAb levels and 15 healthy blood donors as controls were enrolled into this study from January 2011 to December 2012. At least 5 mL serum sample was obtained from each participant. Serum TgAbs and thyroid peroxidase antibodies (TPOAb) were analyzed using an electrochemiluminescence immunoassay and a Cobas e601 analyzer for signal detection (Roche Diagnostics, reference range 0–115 IU/mL for TgAb, 0–34 IU/mL for TPOAb). In the HT group, the TgAb levels ranged from 200 to 4000 IU/mL, with a number of samples exceeding 4000 IU/mL. As we found in our previous study, glycosylation of TgAb IgG might be correlated with TgAb IgG levels in the serum of HT patients (19). Therefore, HT patients in the present study were divided into two subgroups according to their serum TgAb levels. The 17 HT patients with TgAb levels exceeding 4000 IU/mL were classified as high TgAb level group (hHT), while the remaining patients exhibiting moderate TgAb levels (200 to 1500 IU/mL) were categorized as the medium level group (mHT). All HT patients previously diagnosed with hypothyroidism received levothyroxine treatment. Prior to this study, all these patients were verified to be euthyroid, ie, all showed normal thyroid function. Detailed information is provided in Table 1. In the control group, all individuals were euthyroid, thyroid autoantibody-negative, and were confirmed to have no past or family history for thyroid diseases. All serum samples collected were kept at −80°C until further use.

**Table 1.** Demographic Data and TgAb Levels in the HT and Control Groups

Group	Age^a^ (y)	Sex (F/M)	TgAb^b^ levels
HT (n = 32)	45.97 ± 16.46	31/1	3072.0 (428.2–4000)^c^
mHT (n = 15)	42.07 ± 15.92	14/1	417.9 (360.9–708.2)^d^
hHT (n = 17)	49.41 ± 16.62	17/0	All > 4000
Con (n = 15)	42.47 ± 12.60	13/2	10.0 (10.0–17.7)

Abbreviations: HT, Hashimoto's thyroiditis; mHT, Hashimoto's patients with medium TgAb levels; hHT, Hashimoto's patients with high TgAb levels; Con, healthy blood donors.

a Mean±sd.

b Median (interquartile range); normal range of TgAb levels: 0–115 IU/mL.

c *P* < .01 vs Con.

d *P* < .01 vs hHT.

### Affinity purification of total serum IgG

Serum samples were initially filtered three times using 0.20 μm Minisart filters (Sartorius Stedim Biotech). IgG was purified using Hitrap Protein G HP (5 mL) and AKTA purifier (both GE Healthcare) according to the manufacturer's instructions. Briefly, serum sample was pumped after the column was equilibrated with five column volumes of binding buffer (0.02 M Tris, pH 7.2). Unbound protein was removed by washing with five column volumes of binding buffer. Bound IgG was eluted with five column volumes of elution buffer (0.1 M glycine, pH 2.7) and immediately neutralized with 0.2 M Tris (pH 9.0). The IgG solution was dialyzed with 0.01 M phosphate buffered saline (PBS) at 4°C for at least 16 h, and then ultrafiltered using Amicon Ultra Centrifugal Filters (Merk KGaA). Concentrated IgG samples were stored at −80°C until further use.

### Affinity purification of TgAb IgG

To purify TgAb IgG from the total IgG samples, cyanogen bromide-activated Sepharose 4B (Sigma-Aldrich) was conjugated to human thyroglobulin (hTg) (Calbiochem). In this experiment, 2 g of cyanogen bromide-activated resin was washed and left to swell in aliquots of 400 mL cold hydrochloric acid (1 mM). 5 mL of hTg (1 mg/mL) dissolved in coupling buffer (0.1 M sodium bicarbonate buffer containing 0.5 M sodium chloride, pH 8.3) was incubated with approximately 5 mL Sepharose 4B gel for 2 h at room temperature. More than 95% of the hTg was found to have bound to the Sepharose gel. Unbound hTg was removed using the coupling buffer, and the remaining active groups on the Sepharose 4B were blocked with 0.2 M glycine (pH 8.0) for 2 h at room temperature. The Sepharose gel-bound hTg was then washed three times with high and low pH buffer solutions, consisting of either coupling buffer (pH 8.3) or 0.1 M acetate buffer containing 0.5 M sodium chloride (pH 4.0). Then, the Sepharose gel-bound hTg was transferred to a column XK16/20 with two adapters (GE Healthcare) and allowed to precipitate by gravity to the bottom of column for about 20 min. The column was packed according to the instructions of the manufacturer.

TgAb IgG was further purified using a similar process to that used for IgG purification. The binding buffer was 0.01 M PBS (pH 7.4) and the elution buffer was 0.1 M Glycine containing 0.5 M sodium chloride (pH 2.7). Protein concentrations were determined using a BCA protein assay kit (Kangweishiji) and all TgAb IgG samples were stored at −80°C until further use.

### Glycosylation profiles analysis of purified TgAb IgG by MALDI-QIT-TOF-MS/MS

500 μg mixed TgAb IgG from each group dissolved in 0.05 M ammonium bicarbonate buffer was treated successively with 10 μg/μL dithiothreitol solution for 60 min at 37°C, 12 μg/μL iodoacetamide in the dark at room temperature for 1 h, and trypsin at 37°C overnight. Three drops of 5% acetic acid were added to the sample after boiling for 2 min, and then it was desalted using C18-Sep-Pak column (Waters Associates). After the sample was dissolved again in ammonium bicarbonate buffer, it was incubated with N-Glycosidase F (New England Biolabs Ltd.) at 37°C for 20 h. The sample was then lyophilized, dissolved in 5% acetic acid, and desalted again. Sodium hydroxide slurry in dimethyl sulfoxide and methyl iodide were added to lyophilized sample and then vortexed at room temperature for 20 min. Afterwards, chloroform and D.I. water were added to the sample, mixed thoroughly and centrifuged, and the upper aqueous layer was removed. This procedure was repeated three times. The chloroform layer was dried off, and the sample was dissolved in 1:1 methanol: D.I. water, then again desalted and lyophilized. The derivatized glycans were dissolved in 10 μL methanol. 1 μL glycans was mixed with 1 μL 2,5-dihydroxybenzoic acid solution (5 mg/mL 2,5-dihydroxybenzoic acid in 50% acetonitrile containing 0.1 M sodium chloride) for mass spectrometric analysis. The sample matrix mixture was spotted onto a MALDI-TOF plate, and allowed to dry at room temperature. The MALDI data were obtained at positive mode with power setting 70, mass range from 500 to 5000 U, 100 shots per sample using the MALDI-QIT-TOF Mass Spectrometer (Shimadzu Axima Resonance).

### Detection of glycosylation of purified TgAb IgG using high-density lectin microarray

The lectin microarray used in our study was kindly provided by Professor Tao (Shanghai Jiao Tong University, China), and was designed according to a procedure published previously (20, 22). The lectin microarray system consists of a panel of 94 lectins. The lectin specificities and sources have been reported in their studies (20, 22). Prior to use, the surface of the microarray was first blocked for 1 h at room temperature by immersion in 0.05 M ethanolamine in a borate buffer (pH 8.0). The slide was then washed and dried by spinning at 500 *g* for 5 min. 10 μg purified TgAb IgG from each participant was resuspended in 200 μL PBST buffer (PBS buffer with 0.05% TWEEN-20). The samples were applied to the microarray and incubated at room temperature for 2 h. In order to oxidize the sugar groups, 2 μg/mL hTg conjugated with Lightning-Link Rapid Cy5 (Innova Biosiences) was mixed with 0.02 M sodium periodate at 4°C for 1 h in the dark. Then, 200 μL of 2 μg/mL oxidized hTg-Cy5 conjugate was hybridized with the microarray for 1 h. After three washes with PBST buffer and two washes with D.I. water, the array was dried by spinning at 500 g for 5 min, and scanned using a Lux Scan 10K-A scanner (CapitalBio Corporation) at a wavelength of 647 nm and a photomultiplier tube setting of 800. The slide images were converted to numerical format for analysis. The signal-to-noise ratio (S/N) (the medium intensity of the spot foreground relative to the background) of each lectin spot was calculated.

### Statistical analysis

Statistical analysis was performed using the SPSS statistics package (version 17.0). Quantitative data were presented as means ± SD (for normally distributed data) or median and quartiles (for non-normally distributed data) as appropriated. Differences of quantitative parameters between two groups were assessed using Student's *t-*test (for normally distributed data) or Mann-Whitney *U-*test (for non-normally distributed data) as appropriate. A *P* value under .05 was considered statistically significant.

## Results

### Demographic data of participants

As shown in Table 1, significant differences in age and gender distribution existed neither between the HT and control group, nor between the mHT and hHT group. The average serum TgAb levels were significantly higher in the HT group compared with the control group (*P* < .01), and they were also significantly higher in the hHT group compared with the mHT group (*P* < .01). Eleven patients in the mHT group and 15 patients in the hHT group were found to test positive for TPOAb. The average serum TPOAb levels were significantly higher in the HT group compared with those in the control group (*P* < .01). However, no significant difference was measured between the mHT group [200.7(120.4–600)] IU/mL and the hHT group [508.7(156.7–600)] IU/mL.

### Glycosylation profile analysis of purified TgAb IgG by MALDI-QIT-TOF-MS/MS

**Figure 1.**
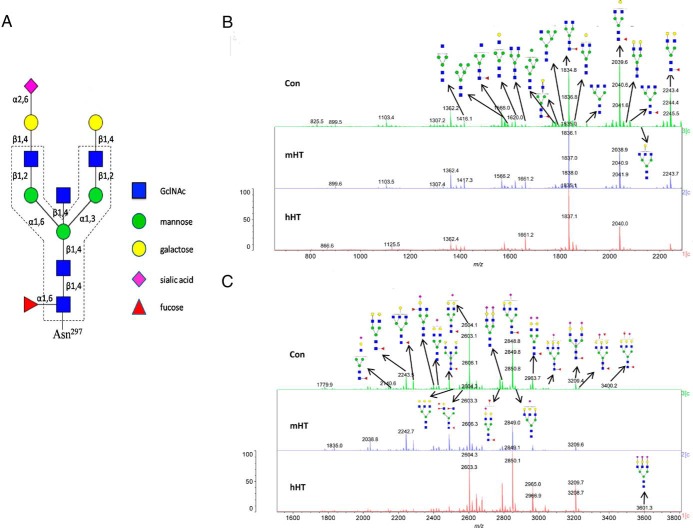
A, Schematic depiction of a monosialylated N-glycan chain linked to Asn^297^ of IgG. The dashed line indicates the conserved core heptasaccharide. The core fucose, terminal galactose and sialic acid, bisecting N-acetylglucosamine (GlcNAc) showed great heterogeneity. B and C, glycosylation profiles of purified TgAb IgG from healthy controls (Con, n = 15), HT patients with medium TgAb levels (mHT, n = 15, TgAb 200–1500 IU/mL), and HT patients with high TgAb levels (hHT, n = 17, TgAb ≥ 4000 IU/mL) by MALDI-QIT-TOF-MS/MS at mass/charge 700–2300 (B) and 1500–3800 (C).

IgG-associated N-glycans have a conserved heptasaccharide core that consists of N-acetylglucosamine (GlcNAc) and mannose (Man), while they show great heterogeneity due to the variations in terminal galactose (Gal), sialic acid (also N-acetyl neuraminic acid, abbreviated as Neu5Ac), core fucose (Fuc), and bisecting GlcNAc (7) (Figure 1A). The carbohydrates, named glycoforms, of each mixed TgAb IgG sample from the mHT, hHT, and control groups were measured by MALDI-QIT-TOF-MS/MS, and its analysis revealed that the glycosylation profiles of TgAb IgG from the mHT, hHT, and control groups were extremely similar (Figure 1, B and C). The first four of the most intense peaks were at the mass/charge (m/z) positions 1835.9, 2040.0, 2605.3, and 2850.4. The first three of those four represent three biantennary glycoforms, namely G0F (core fucosylated, no terminal galactose), G1F (core fucosylated, one terminal galactose without sialic acid), and G2SF (core fucosylated, two galactose with one terminal sialic acid). The last peak represents one complex triantennary glycoform (Figure 1, B and C). A total of 34 N-linked glycoforms were identified in the TgAb IgG, shown in Supplemental Table 1.

### Detectable lectins and glycans of purified TgAb IgG in lectin microarray

In order to analyze the glycans present on purified TgAb IgG from the mHT, hHT, and control groups, we utilized a lectin microarray. To prevent nonspecific binding of hTg to lectins, the hTg-Cy5 conjugates were treated with sodium periodate, which resulted in the oxidization of the associated glycans. The detectable lectin signals were defined as (1) (S/N of lectin spots with sample) − (S/N of lectin spots with PBS) > 0.5; (2) (S/N of lectin spots with sample)/(S/N of lectin spots with PBS) > 1.5 for over 50% samples from at least one group. Lectins conforming to the first condition and those conforming to the second condition overlapped substantially, with a total number of 8 detectable lectins (Table 2).

**Table 2.** Detectable Lectins in the Lectin Microarray

Full name	Abbrev.	Ref.	Specificity	Source
Lens Culinaris Agglutinin	LCA	5	Man	3
Morniga M Lectin	MNA-M	4	Manα	4
Vicia fava Lectin	VFA	1	Manα	4
Phaseolus vulgaris Lectin	PHA-L	2	Gal (β1–4)GlcNAc (β1–2)Man	1
Polyporus squamosus	PSA	1	Neu5Ac (α2–6)Gal (β1–4)GlcNAc, Neu5Ac (α2–6)Gal (β1–4)Glc	2
Sambucus nigra I	SNA-I	1	Neu5Ac (α2–6)Gal (β1–4)GlcNAc, Neu5Ac (α2–6)Gal (β1–4)Glc	4
Sambucus nigra	SNA	2	Neu5Ac (α2–6)Gal, Neu5Ac (α2–6)GalNAc	2
Lens culinaris Lectin	LcH	3	complex (Man/GlcNAc core with Fucα1–6)	4

Detectable lectins were defined as (1) (S/N of each lectin spot with sample) − (S/N of lectin spot with PBS) > 0.5; (2) (S/N of lectin spot with sample)/(S/N of lectin spot with PBS) > 1.5 for over 50% samples from at least one group. The carbohydrate specificities of lectins were obtained using five different references: 1, the database of the Consortium for Functional Glycomics; 2, Hsu et al. (21); 3, Wearne et al. (36); 4, the lectin carbohydrate specificity chart courtesy of EY laboratories; 5, manual associated with the lectin purchased from EY laboratories or Vector. The lectins used in this study were obtained from four different sources: 1, Molecular Probes Inc; 2, Irwin J. Goldstein's group; 3, Vector laboratories Inc; 4, EY Laboratories Inc. Sugar abbreviations: Fuc, Fucose; Gal, Galactose; GalNAc, N-Acetylgalactosamine; Glc, Glucose; GlcNAc, N-Acetylglucosamine; Man, Mannose; Neu5Ac, N-Acetylneuraminic acid (sialic acid).

### Amounts of glycans present on purified TgAb IgG differ between HT patients and healthy controls

**Figure 2.**
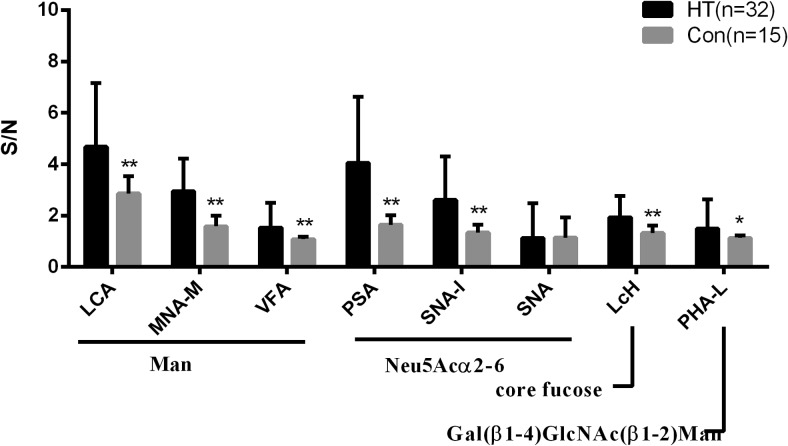
S/N of 8 detectable lectins for purified TgAb IgG from healthy controls (Con, n = 15) and HT patients (n = 32). Binding specificities were LCA, MNA-M, VFA for mannose (Man); PSA, SNA- I and SNA for terminal Neu5Acα2–6 (sialic acid); LcH for core fucose; PHA-L for Gal(β1–4)GlcNAc(β1–2)Man. **, *P* < .001; *, *P* < .01.

Our results revealed the following changes of glycosylation on TgAb IgG for HT patients relative to controls (Figure 2): (1) increased mannose evidenced by increased binding to Lens Culinaris Agglutinin (LCA), Morniga M Lectin (MNA-M), and Vicia fava Lectin (VFA) (specificity as shown in Table 2) (all *P* < .001); (2) increased terminal Neu5Ac (sialic acid), as indicated by the increased binding to polyporus squamosus (PSA) and sambucus nigra I (SNA-I) (both *P* < .001); (3) increased core fucose detected by increased binding to Lens culinaris Lectin (LcH) (*P* < .001); (4) increased Gal(β1–4)GlcNAc(β1–2)Man glycans detected by increased binding to Phaseolus vulgaris Lectin (PHA-L) (*P* = .00283). There was no significant difference of Sambucus nigra (SNA) between the HT group and the control group.

### Amounts of glycans on purified TgAb IgG differ between the mHT and hHT subgroups

**Figure 3.**
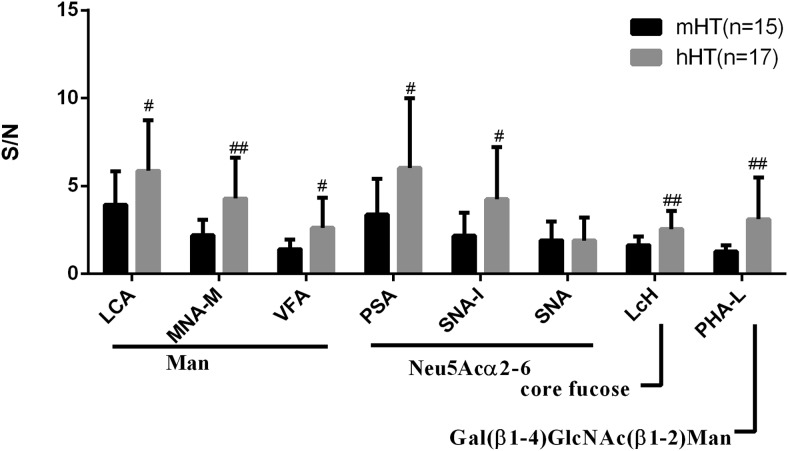
S/N of 8 detectable lectins for purified TgAb IgG from HT patients with medium TgAb levels (mHT, n = 15, TgAb 200–1500 IU/mL) and with high TgAb levels (hHT, n = 17, TgAb ≥ 4000 IU/mL). Binding specificities were LCA, MNA-M, VFA for mannose; PSA, SNA-I, and SNA for terminal Neu5Acα2–6 (sialic acid); LcH for core fucose; PHA-L for Gal(β1–4)GlcNAc(β1–2)Man. ##, *P* < .01; #, *P* < .05.

As shown in Figure 3, the S/N ratios of LCA, MNA-M, VFA, PSA, SNA-I, LcH, and PHA-L for TgAb IgG from the hHT group were all significantly greater than those from the mHT group: *P* = .0312 (LCA), *P* = .0114 (VFA), *P* = .0025 (MNA-M), *P* = .0151 (SNA-I), *P* = .0234 (PSA), *P* = .0032 (LcH), *P* = .0057 (PHA-L), which indicated that there was a greater amount of mannose, terminal sialic acid, core fucose, and Gal(β1–4)GlcNAc(β1–2)Man on TgAb IgG from the hHT group than that from the mHT group.

## Discussion

Over the past decade, numerous studies have explored the various structures, as well as the biological and clinical roles of IgG glycosylation. HT is an autoimmune thyroid disease, and TgAbs are commonly found at high titers in the sera of HT patients (2). Studies aimed at understanding the mechanisms of loss of self-tolerance in HT have found that the titers and epitopes of serum TgAbs in HT patients differ from those in healthy individuals (4, 23, 24). As protein glycosylation is one of major post-translational modifications, and because of its important role in the Fc-mediated biological function of IgGs, studying the differential expression of TgAb IgG glycosylation patterns between HT patients and healthy controls will help to understand the biologic role of TgAb IgG in the pathogenesis of HT.

It was shown previously that the N-linked glycans at the highly conserved asparagine 297 residue in the Fc region of IgG are mainly biantennary complex-type structures with a core heptasaccharide GlcNAc(β1–2)Man(α1–6)(GlcNAc(β1–2)Man(α1–3))Man(β1–4)GlcNAc(β1–4) GlcNAc (8). This can vary by the addition of fucose to the core GlcNAc, by addition of GlcNAc to the bisecting mannose, or by extending the arms with galactose and sialic acid (8). Our study presented here focused on the glycosylation of the thyroid specific antibody. Because the amount of purified TgAb IgG from any one participant is relatively small, we used a mixture of TgAb IgG from all participants in each group, with the glycoforms of 500 μg TgAb IgG of the mHT, hHT, and control groups being profiled separately by MALDI-QIT-TOF-MS/MS. A total number of 34 glycoforms were found on TgAb IgG in our study. The results showed that there was no significant difference in glycosylation profiles of purified TgAb IgG between Hashimoto's patients and healthy controls. The most abundant structures of TgAb IgG were fucosylated in a biantennary manner, either containing one sialic acid or none.

Although MALDI-QIT-TOF-MS/MS can reveal molecular details of glycans, it requires a significant investment of time and an abundant amount of purified proteins. In contrast, the lectin microarray at hand was shown to facilitate the extraction the glycan structure information of glycoproteins at the nanogram level (20), in both a high-throughput and a sensitive fashion (25). Therefore, it was used to further compare the amounts of different glycans of TgAb IgG between the HT group and control group. Our results showed that the glycosylation levels of TgAb IgG differed between the HT group and control group, and that there were greater amounts of mannose, terminal sialic acid, core fucose, and Gal(β1–4)GlcNAc(β1–2)Man associated with TgAb IgG in HT patients. Changes in the patterns as well as levels of IgG glycosylation have been reported for many different autoimmune diseases, including primary Sjogren's syndrome, ANCA-associated systemic vasculitis, and myositis syndromes (13–15). Importantly, as several studies have shown, the changes of IgG glycosylation are correlated with the severity of rheumatoid arthritis, a fact that supports the idea that glycosylation changes play an important role in inflammatory processes in rheumatoid arthritis (16, 26). Considering the important role of glycosylation in the structure and function of IgG, we speculated that the changes in TgAb IgG glycosylation patterns in HT patients might affect the structure and function of TgAb, especially its Fc-mediated functions, such as ADCC. In conclusion, the altered TgAb IgG glycosylation patterns observed here might contribute to the pathogenesis of HT. However, further studies are required to test the precise connection between TgAb IgG glycosylation and HT pathogenesis.

An earlier study by Wang (27) demonstrated that cytokines in the microenvironment of B cells can not only determine the subsequent differentiation of B cells into antibody-secreting cells such as interleukin-2 (IL-2) and IL-10, but also regulate the glycosylation of antibodies produced, such as interferon-γ and IL-21. TgAb are produced by lymphocytes infiltrated in the thyroid tissue (28). It was reported that there is an imbalance of Th1/Th2 cells and increased Th17 cells in the sera of HT patients, and the cytokine profiles produced in HT patients are different from those in healthy donors, with increased levels of cytokines such as interferon-γ, IL-2, and/or IL-17 (29–32). We speculate that the different cytokine patterns in the thyroid tissue of HT patients might contribute to the differentiation of different subsets of plasma cells, and subsequently affect the different levels of glycosylation on TgAb IgG. Further studies will be required to investigate the role of cytokines in the thyroid tissue on TgAb glycosylation in greater detail.

Glycosylation is an enzyme-mediated post-translational process (33), and changes in glycans therefore reflect altered enzyme expression levels and/or activities (34). Thus, the different patterns of TgAb IgG glycosylation in HT patients and healthy controls can be attributed to different levels or activities of corresponding enzymes in different subsets of plasma cells (35). Because the observed levels of several kinds of glycans on TgAb IgG were higher in HT patients than those observed for healthy controls in our study, we would argue that change of one glycosylation-related enzyme might not be sufficient to explain the extensive differences. Further studies on the expression pattern of these enzymes in plasma cells should shine light on the underlying mechanisms in further detail.

In addition, our present study showed that there were greater amounts of mannose, terminal sialic acid, core fucose, and Gal(β1–4)GlcNAc(β1–2)Man on TgAb IgG from the hHT than that from mHT. This indicates that the glycosylation pattern of TgAb might be related to the serum TgAb levels. In contrast, our previous study, in which we used elderberry lectin named SNA, showed that the sialic acid content on TgAb was negatively correlated with the serum TgAb IgG levels in all the patients of different thyroid diseases (19). This may be due to the different sources of SNA or different methods or different experimental material, with the former using serum, and our study presented here using purified TgAb IgG.

In conclusion, our present study provides for the first time evidence that the glycosylation levels of TgAb IgG in HT patients is elevated, and that the glycosylation patterns are altered relative to TgAb IgG found in healthy donors. Thus, our study provides new clues that should allow for more detailed exploration of the role of TgAb in the pathogenesis of HT in the future.
